# MLR-1023 Treatment in Mice and Humans Induces a Thermogenic Program, and Menthol Potentiates the Effect

**DOI:** 10.3390/ph14111196

**Published:** 2021-11-22

**Authors:** Candida J. Rebello, Ann A. Coulter, Andrew G. Reaume, Weina Cong, Luke A. Cusimano, Frank L. Greenway

**Affiliations:** 1 Clinical Trials Unit, Pennington Biomedical Research Center, Baton Rouge, LA 70808, USA; Candida.Rebello@pbrc.edu (C.J.R.); Ann.Coulter@pbrc.edu (A.A.C.); 2 Melior Discovery Inc., 860 Springdale Drive, Exton, PA 19341, USA; areaume@meliorpharma.com (A.G.R.); vcong@meliordiscovery.com (W.C.); 3 Cusimano Plastic and Reconstructive Surgery, 5233 Dijon Dr, Baton Rouge, LA 70808, USA; drc@drcmdplasticsurgery.com

**Keywords:** MLR-1023, menthol, beige fat, UCP1, thermogenesis, insulin sensitivity, body weight, blood glucose

## Abstract

A glucose-lowering medication that acts by a different mechanism than metformin, or other approved diabetes medications, can supplement monotherapies when patients fail to meet blood glucose goals. We examined the actions underlying the effects of an insulin sensitizer, tolimidone (MLR-1023) and investigated its effects on body weight. Diet-induced obesity (CD1/ICR) and type 2 diabetes (db/db) mouse models were used to study the effect of MLR-1023 on metabolic outcomes and to explore its synergy with menthol. We also examined the efficacy of MLR-1023 alone in a clinical trial (NCT02317796), as well as in combination with menthol in human adipocytes. MLR-1023 produced weight loss in humans in four weeks, and in mice fed a high-fat diet it reduced weight gain and fat mass without affecting food intake. In human adipocytes from obese donors, the upregulation of Uncoupling Protein 1, Glucose (UCP)1, adiponectin, Glucose Transporter Type 4 (GLUT4), Adipose Triglyceride Lipase (ATGL), Carnitine palmitoyltransferase 1 beta (CPT1β), and Transient Receptor Potential Melastin (TRPM8) mRNA expression suggested the induction of thermogenesis. The TRPM8 agonist, menthol, potentiated the effect of MLR-1023 on the upregulation of genes for energy expenditure and insulin sensitivity in human adipocytes, and reduced fasting blood glucose in mice. The amplification of the thermogenic program by MLR-1023 and menthol in the absence of adrenergic activation will likely be well-tolerated, and bears investigation in a clinical trial.

## 1. Introduction

Worldwide, diabetes affected 463 million people in 2019, and is expected to reach 700 million people by 2045 [1]. In the United States (US), an estimated 10.5% of the population have diabetes, and more than one-third have prediabetes [2]. The severe acute respiratory syndrome coronavirus 2 (SARS-CoV-2) pandemic (COVID-19) exposed the vulnerability of people with diabetes, especially those with poorly controlled blood glucose [3]. Emerging evidence relating to post-acute infectious consequences of COVID-19 includes new diagnoses and worsening control of existing diabetes mellitus [4]. In patients with pre-existing type 2 diabetes, blood glucose control is associated with a reduced risk of all-cause mortality and adverse outcomes from COVID-19 [3].

Stemming from its efficacy, lack of associated hypoglycemia, low cost, and potential to induce small reductions in body weight, metformin is recommended as the first line of therapy for type 2 diabetes [5,6]. Over time, most patients are unable to maintain glycemic control with metformin monotherapy. It is estimated that the five-year incidence of failure to control blood glucose is 20–50% in individuals prescribed metformin alone [7,8,9,10]. In the UK Prospective Diabetes Study, only 50% of patients with newly diagnosed diabetes maintained recommended glycemic goals with monotherapy after three years, which reduced to 25% after nine years [9]. Undoubtedly, most patients with diabetes will require an addition to metformin.

Tolimidone; CP-26154; 2(1H)-Pyrimidinone, 5-(3-methylphenoxy) was originally created and developed by Pfizer Inc. to treat gastric ulcers, but was discontinued in phase II testing due to a lack of efficacy. However, Tolimidone was well-tolerated, making it imminently suitable for drug repositioning. Phenotypic screening to identify otherwise unpredicted uses helped to identify new biology associated with Tolimidone. Activity in an oral glucose tolerance test and a reduction in weight gain in animals exposed to a high-fat diet were among the metabolic outcomes identified [11]. Renamed MLR-1023, the repurposed drug was observed to have a potent effect on lowering blood glucose by selectively activating Lyn kinase [12,13]. MLR-1023 exhibits an EC_50_ of 63 nM for Lyn kinase activation [13]. In knockout mice lacking Lyn kinase, MLR-1023 does not have any glucose lowering effect [13]. The activation of Lyn kinase reduces blood glucose by potentiating insulin receptor activation and the insulin receptor substrate-phosphatidylinositol-3 (IRS-PI3K) insulin signaling pathway, thereby mitigating insulin receptor deficiencies in muscle and adipose tissue [14]. In mice, MLR-1023 increases insulin sensitivity, measured using the gold standard hyperinsulinemic euglycemic clamp test, and does not cause hypoglycemia [13,15]. Importantly, in a randomized double-blinded placebo-controlled clinical trial, 100 mg/day of MLR-1023 reduced fasting plasma glucose in four weeks [15].

Insulin resistance plays a central role in the pathogenesis of type 2 diabetes and the macroangiopathic complications of the disease [16]. The thiazolidinedione (TZD) class of drugs, peroxisome-proliferator-activated receptor gamma (PPARγ) activators, was approved more than 20 years ago, and is the only insulin-sensitizing medication for the treatment of type 2 diabetes [17]. Glucose-lowering medications that act by increasing insulin sensitivity do not induce hypoglycemia [17]. Therefore, TZDs are not associated with an increased risk of hypoglycemia, but the clinical use of TZDs has been hindered by safety issues [17]. The TZD class of drugs induces fluid retention, and the ensuing adverse metabolic outcomes include bone fractures and a 70% increased risk of congestive heart failure [18,19,20]. Furthermore, TZD treatment causes weight gain and increases the risk of bladder cancer [7,21,22].

In response to a glucose challenge, metformin lowers blood glucose in both wild-type and Lyn kinase knockout animals, demonstrating that MLR-1023 and metformin administration affect glucose concentrations by different mechanisms. Moreover, the glucose-lowering response produced with MLR-1023 is equivalent in magnitude to that of metformin [12,13]. In chronically treated mice, MLR 1023 elicits a dose-dependent and durable glucose-lowering effect, a reduction in glycated hemoglobin (HbA1c) levels, and the preservation of pancreatic β-cells. The magnitude of the effect is equivalent to that observed with rosiglitazone, which is a TZD, but with a faster onset of action and without causing weight gain [13]. These results support a glucose-lowering effect of MLR-1023, which is distinct from metformin and TZDs [12,13].

In adipocytes, Lyn kinase promotes the phosphorylation and activation of IRS-1. The resulting upregulation in insulin signaling and glucose transporter type 4 (GLUT4) translocation improves insulin sensitivity [14,23]. Furthermore, adiponectin is secreted by adipocytes, and it improves insulin sensitivity in the liver and muscle. The resulting metabolic effects include reductions in hepatic glucose production, de novo lipogenesis, and triglyceride storage [24]. Adiponectin also increases the expression of uncoupling protein 1 (UCPI) in brown adipose tissue and reduces body weight in mice without affecting food intake, indicating increased thermogenesis [25].

Several approaches to exploiting the therapeutic value of adiponectin are being investigated [16]. Similarly, transient receptor potential melastin (TRPM8) is a cold sensor present in brown and white adipose tissue that can trigger UCP1-induced thermogenesis [26,27]. Menthol is a cooling agent from the mint plant and a potent TRPM8 agonist [28]. Studies in rodents and in vivo human models suggest a role for menthol in the stimulation of brown adipose tissue activity mediated by TRPM8 [26,29]. We hypothesized that MLR-1023 acts in adipose tissue to stimulate the secretion of adipokines and the regulation of genes related to thermogenesis and insulin sensitivity, and that in combination with menthol the effect would be potentiated.

The goal of this study was to clarify the molecular mechanisms underlying the insulin-sensitizing effects of MLR-1023 and investigate its effects on body weight. We observed that MLR-1023 promotes weight loss in humans, and prevents weight gain in mice fed a high-fat diet. The effects on body weight in mice were not due to a reduction in food intake, and suggest that MLR-1023 increases energy expenditure. Our studies in human adipocytes from obese donors demonstrated that MLR-1023 increased the expression of genes involved in thermogenesis and insulin sensitivity, suggesting a conversion to the brown/beige phenotype. Notably, we observed that TRPM8 was significantly upregulated. Furthermore, MLR-1023 potentiated the menthol-induced activation of TRPM8.

## 2. Results

### 2.1. MLR-1023 Reduces Weight Gain in Mice Fed a High-Fat Diet without Affecting Food Intake

**Figure 1:**
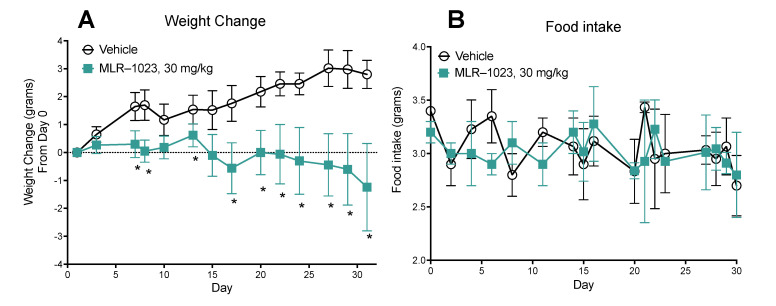
Effect of 30 mg/kg of MLR-1023 twice daily on body weight and food intake in CD1/ICR mice. The mice were weighed daily, and food intake was monitored every one to four days. (**A**) Weight change from day 0 to day 31 (**B**) Food intake from day 1 to day 31. N = 6 per group. Values are mean ± SEM. * *p* < 0.05.

**Figure 2:**
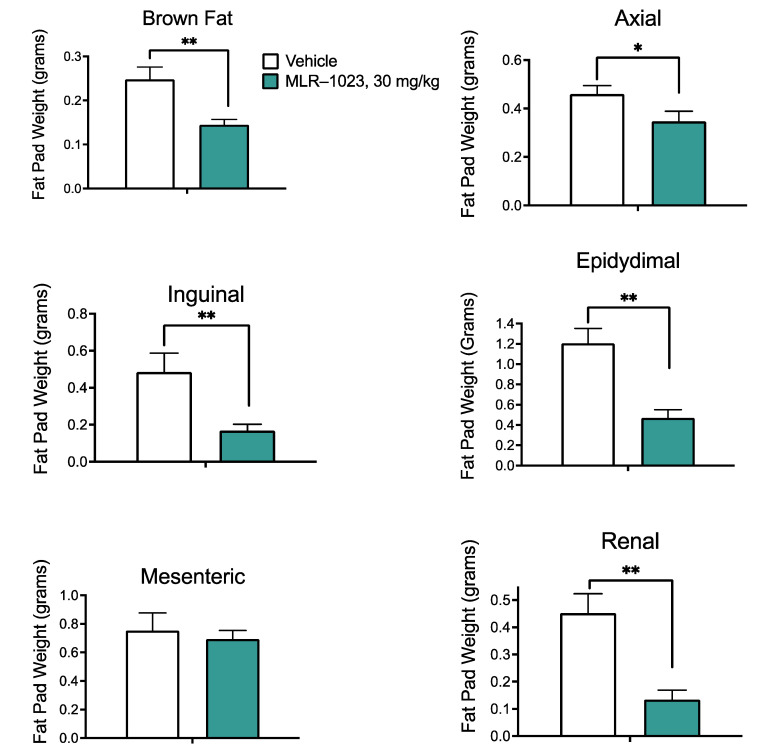
Effect of 30 mg/kg of MLR-1023 twice daily on fat mass in CD1/ICR mice. Brown, axial, inguinal, epididymal, mesenteric, and renal fat pads were dissected and weighed at the end of the study (day 31). N = 6 per group. Values are mean ± SEM. * *p* < 0.05. ** *p* < 0.01.

**Figure 3:**
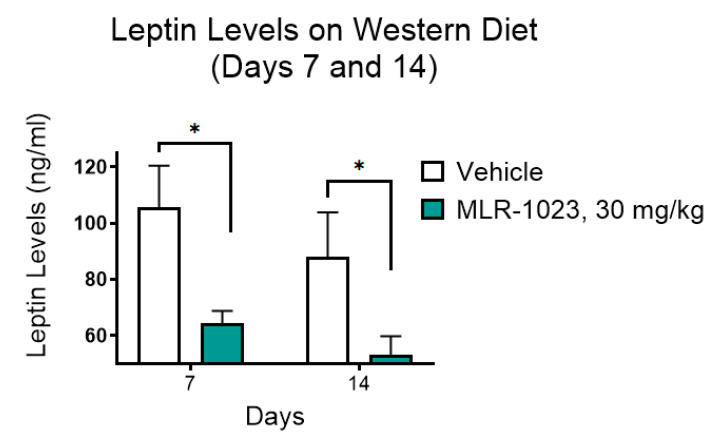
Effect of 30 mg/kg of MLR-1023 twice daily on blood leptin concentrations in CD1/ICR mice. The mice were dosed intraperitoneally one hour prior to being bled by a retro-orbital eye bleed on days 7 and 14 after the initiation of the study. Leptin concentrations were measured on days 7 and 14. N = 6 per group. Values are mean ± SEM. * *p* < 0.05.

We examined the effect of a 30-day treatment with MLR-1023 (30 mg/kg) on body weight in CD1/ICR mice fed a high-fat diet, designed to mimic the typical diet consumed in North America. There was a statistically significant difference in body weight change by day seven (MLR-1023: 0.3 ± 0.5 g; vehicle: 1.7 ± 0.5; and *p* = 0.04). At the end of the study, mice treated with MLR 1023 lost weight compared to the vehicle-treated group (MLR 1023: −1.2 ± 1.6; vehicle: 2.8 ± 0.5; and *p* = 0.04, Figure 1A). Interestingly, food intake was not altered in the mice treated with MLR-1023 (Figure 1B). The results suggest that MLR-1023, administered at 30 mg/kg twice daily, increases energy expenditure. The reduction in body weight with MLR-1023 treatment was accompanied by significant decreases in the mass of brown (*p* = 0.003), axial (*p* = 0.033), inguinal (*p* = 0.007), epidydimal (*p* = 0.001), and renal (*p* = 0.001) fat pads (Figure 2). Furthermore, compared to the vehicle-treated group, plasma leptin concentrations reduced in the MLR-1023-treated group (day seven: *p* = 0.012; day 14: *p* = 0.04, Figure 3). Leptin is released by adipocytes in proportion to fat mass. Therefore, the reduction in leptin concentrations is consistent with the decrease in adiposity that occurred with MLR-1023 treatment.

### 2.2. MLR-1023 Increases the Expression of Genes for Thermogenesis and Glucose Utilization in Human Adipocytes

**Figure 4:**
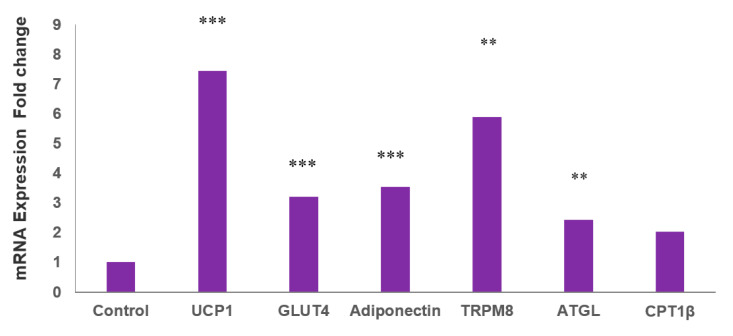
mRNA expression in human adipocytes (HASCs) treated with MLR-1023. qRT-PCR assays were conducted in duplicates following MLR-1023 (60 µM) treatment of HASCs from three obese donors (four technical replicates each) for seven days compared to untreated adipocytes (control). The results are presented as mean ± SE, ** *p* ≤ 0.01. *** *p* ≤ 0.001 compared to the control.

The durability of the insulin-sensitizing effect of MLR-1023 is consistent with an effect on adipose tissue, which secretes numerous adipokines that increase whole-body glucose utilization [30]. Human adipocytes derived from adult white adipose stem cells (HASCs) were treated for seven days with MLR-1023 at the physiologic C_max_ of 60 µM [13] or the vehicle. The genes for mitochondrial thermogenesis (UCP1) and fat oxidation (carnitine palmitoyltransferase 1β (CPT1β) and adipose triglyceride lipase (ATGL)) were significantly upregulated (Figure 4). UCP1 is a marker for the transformation of white to beige adipocytes [31]. ATGL and CPT1β are the enzymes that catalyze fatty acid release and transport into the mitochondria for oxidation [32,33]. Consistent with the increase in insulin sensitivity that occurs with the induction of a beige thermogenic phenotype in human adipocytes, the expression of the insulin-stimulated glucose transporter, GLUT4, and the insulin sensitizer, adiponectin, increased after MLR-1023 treatment compared to the control (Figure 4).

Expression of TRPM8, the primary cold receptor in sensory neurons [34], also increased after MLR-1023 treatment (Figure 4). In human adipocytes, TRPM8 plays a role in modulating the peripheral response to cold by activating UCP1 expression and the generation of heat [29]. The β-1 adrenergic receptor (β1AR), which is the key receptor that modulates the sympathetic stimulation of thermogenesis in human adipose tissues, was not upregulated [35,36]. These results suggest that MLR-1023 induces a thermogenic program through a molecular pathway that is independent of adrenergic stimulation.

### 2.3. MLR-1023 Enhances TRPM8 Sensitivity to Cold

**Figure 5:**
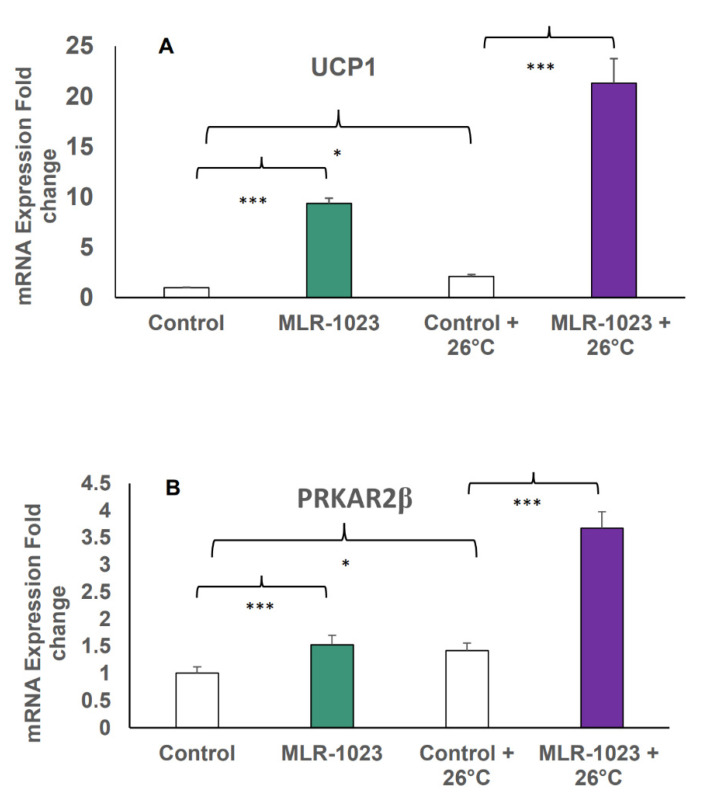
mRNA expression in human adipocytes (HASCs) treated with MLR-1023 and cold stimulation. qRT-PCR assays were conducted in duplicates following MLR-1023 (60 µM) treatment of human adipocytes from three obese donors (four technical replicates each) for seven days compared to untreated adipocytes (control), maintained at 37 °C, or treated as such and maintained at 26 °C for the last 24 h: (**A**) uncoupling protein 1 (UCP1) and (**B**) protein kinase cAMP-dependent type II regulatory subunit beta (PRKAR2β). The results are presented as mean ± SE. * *p* < 0.05, *** *p* ≤ 0.001 compared to the respective control.

To define the role of TRPM8 in the remodeling of human adipocytes induced by MLR-1023, we investigated whether its activity was functionally increased as a potential mechanism by which the genes related to energy expenditure were upregulated. The HASCs were treated with MLR-1023 (60 µM) for seven days or were untreated (control), and maintained at 37 °C. In a parallel experiment, the same treatment and control were administered to HASCs, and they were cooled (placed at 26 °C) for the last 24 h of the seven-day protocol. Control HASCs cooled to 26 °C had an induction in UCP1 expression of over two-fold compared to control cells maintained at 37 °C (*p* < 0.001). The HASCs that were exposed to MLR-1023 for seven days and cooled for 24 h had over 20-fold higher UCP1 and two-fold higher protein kinase cAMP-dependent type II regulatory subunit beta (PRKAR2β) mRNA levels relative to untreated cells maintained at equivalent temperatures (*p* < 0.001). TRPM8 activation of UCP1 occurs in the absence of adrenergic stimulation through the phosphorylation of protein kinase A (PKA) in mouse brown adipose tissue [26]. Thus, in HASCs we observed an upregulation of PRKAR2β, which is the component of PKA that plays an important role in regulating lipolysis and adiposity in humans (Figure 5A,B) [37,38]. These results demonstrate that MLR-1023 amplified the physiologic response to cold stimulation.

### 2.4. MLR-1023 and Menthol Synergistically Induce the Expression of Metabolic Genes

**Figure 6:**
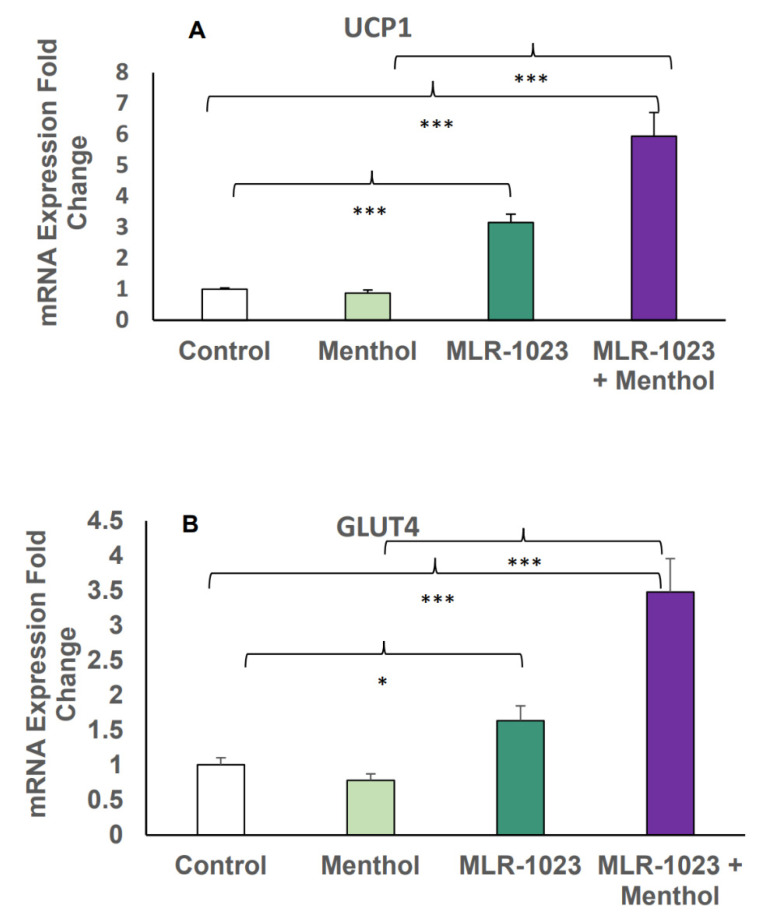
mRNA expression in human adipocytes (HASCs) treated with MLR-1023 and menthol. qRT-PCR assays conducted in duplicates following MLR-1023 (30 µM), menthol (30 µM), and MLR-1023 in addition to menthol (30 µM each) treatment of human adipocytes from three obese donors (four technical replicates each) compared to untreated adipocytes (control): (**A**) uncoupling protein 1 (UCP1) and (**B**) glucose transporter type 4 (GLUT4). The results are presented as mean ± SE. * *p* < 0.05, *** *p* < 0.001 compared to the respective control. MLR-1023 and menthol also exhibit synergy, where the combined effect compared to control is greater than the sum of the effects of MLR-1023 or menthol alone, compared to the control.

**Figure 7:**
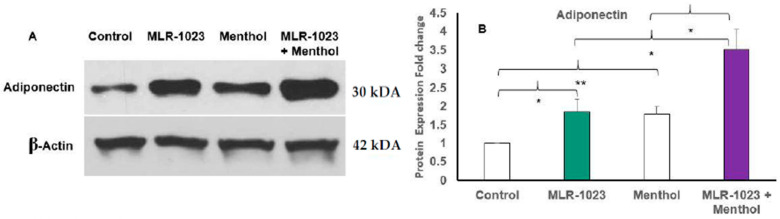
Immunoblotting of human adipocytes (HASCs) treated with MLR-1023 and menthol. HASCs from two obese donors were treated with MLR-1023 (30 µM), menthol (30 µM), and MLR-1023 in addition to menthol (30 µM each) for seven days (four technical replicates each). (**A**) Cellular protein was extracted, and Western blot analysis was used to detect protein levels of adiponectin. (**B**) Densitometric analysis. Values are mean ± SEM. * *p* < 0.05. ** *p* < 0.01.

Menthol is a potent natural compound known to activate TRPM8 and stimulate UCP1-dependent thermogenesis in white adipocytes [26,28]. We examined whether the TRPM8 agonist menthol could further induce the expression of genes involved in thermogenesis and glucose metabolism observed with MLR-1023 treatment. In HASCs, the combination of MLR-1023 and menthol synergistically increased the expression of UCP1 and GLUT4 compared to either MLR-1023 (30 µM) or menthol (30 µM) alone (*p* < 0.001, Figure 6A,B). Furthermore, the protein expression of adiponectin increased in HASCs treated with MLR-1023 and menthol compared to either MLR-1023 or menthol alone (*p* = 0.04, Figure 7A,B).

### 2.5. In Vivo, Menthol Potentiates the Glucose-Lowering Effect of MLR-1023

**Figure 8:**
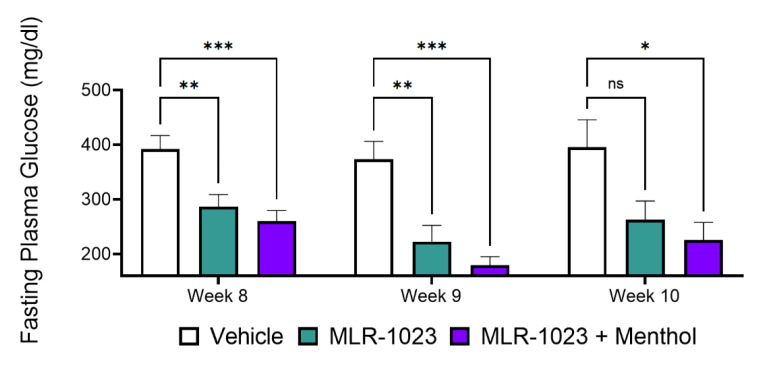
Effect of 100 mg/kg of MLR-1023 alone and in combination with 100 mg/kg of menthol on fasting blood glucose in db/db mice. The mice were treated with MLR-1023, the vehicle, or MLR-1023 + menthol once daily for eight weeks. Mice were followed for two weeks after treatment ended. Lean mice (BKS Cg) were similarly treated with the vehicle. Fasting blood glucose was measured weekly. N = 18 per group for eight weeks and N = 8 for weeks 9 and 10. Values are mean ± SEM. * *p* < 0.05, ** *p* < 0.01, and *** *p* < 0.001. ns = non-significant.

The chronic activity of MLR-1023 and menthol was evaluated in a diabetic mouse model fed a standard chow diet. Treatments were administered once daily to db/db mice for 56 days. Fasting plasma glucose (FPG) was measured weekly during the treatment and for two weeks after the treatment ended. Compared to the vehicle, MLR-1023 (100 mg/kg) reduced FPG at eight weeks (difference: −105.2 ± 33.58 mg/dL, *p* = 0.004) and one week after treatment ended (difference: −150.6 ± 44.55 mg/dL, *p* = 0.005). However, compared to the vehicle, menthol (100 mg/kg) in combination with MLR-1023 produced a significant reduction in FPG after treatment ended (difference: −131.7 ± 31.95 mg/dL, *p* < 0.001) and two weeks later (difference: −169.8 ± 59.95 mg/dL, *p* = 0.017), whereas the reduction by MLR-1023 alone was no longer significant (Figure 8).

### 2.6. MLR-1023 Reduces Body Weight and Fasting Plasma Glucose in Human Subjects

**Figure 9:**
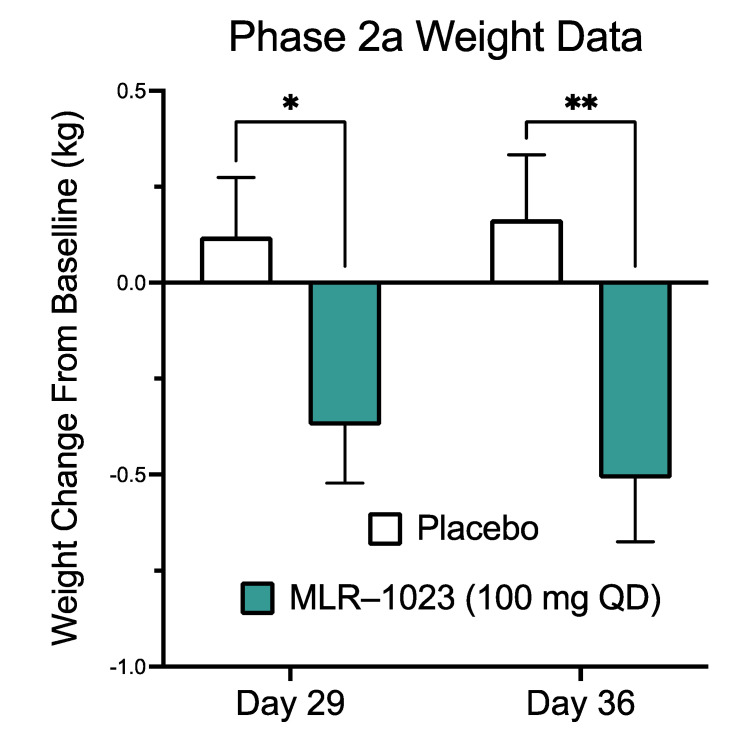
Clinical Trial of MLR-1023 vs. placebo. The study was a randomized double-blinded placebo-controlled parallel-arm phase II trial to investigate the effect of MLR-1023 on body weight in adults with type 2 diabetes. Body weight was measured at baseline, at day 29, and at day 36. Values are mean ± SEM. * *p* < 0.05. ** *p* < 0.01.

In a randomized double-blinded phase IIa clinical trial of MLR-1023 in subjects with type 2 diabetes, we observed a significant weight loss in the US cohort treated for four weeks at 100 mg/day. The baseline characteristics of the subjects are presented in Table 1. Compared to the placebo, weight was reduced in the MLR-1023-treated group (least-squares mean difference: −0.49 ± 0.23 kg, *p* = 0.03) at four weeks and continued to decline for one week after the medication was stopped (difference: −0.67 ± 0.23 kg, *p* = 0.004, Figure 9). Furthermore, FPG (least-squares mean difference: −2.87 ± 0.90 mmol/L, *p* < 0.002) was reduced compared to the placebo at four weeks. These results, in conjunction with preclinical and cell culture studies, suggest that MLR-1023 induces the conversion of adipocytes to the brown/beige phenotype, which is a persisting effect.

## 3. Discussion

The cardiovascular and renal protective effects of SGLT2 inhibitors and GLP-1 receptor agonists have been substantiated, but high costs limit their clinical value [39]. The efficacy and low cost of metformin, coupled with the benefit of weight loss, has rendered it the first line of drug therapy for type 2 diabetes, but gastrointestinal side effects pose a challenge to compliance [40]. Nevertheless, the widespread use of metformin and sulfonylureas, especially in low- and middle-income countries, is a testament to the need for affordable, safe, and efficacious glucose-lowering medications that can supplement monotherapies when patients fail to meet blood glucose goals [41,42,43].

Mouse studies have reported the emergence of beige adipocytes, which are inducible fat cells that appear within white adipose tissue depots [44,45,46,47]. A distinctive feature of beige cells is that they require external stimuli such as cold or β-adrenergic agonists or PPAR activators for the induction of UCP1 [48]. Beige cells have distinct characteristics that are similar to brown adipocytes, including a similar manifestation of UCP1 expression, which is localized in the mitochondrial membrane and acts to uncouple oxidative phosphorylation from ATP production [49]. The resulting dissipation of the electron gradient as heat is a process termed thermogenesis, which increases the utilization of fatty acids for energy expenditure [50]. Pharmacotherapies targeting adipose tissue can confer a long-lasting effect that converts human white adipocytes into metabolically active brown-like cells [51].

In humans, 10 days of cold exposure induces UCP1 in adipocytes, which is accompanied by mitochondrial uncoupled respiration in abdominal subcutaneous white adipose tissue [52]. Mirabegron is a β3-adrenergic agonist approved for the treatment of an overactive bladder, and treatment for 10 weeks induces UCP1 in obese and insulin-resistant individuals. Mirabegron also improves glucose homeostasis, reduces skeletal muscle triglycerides, and increases lipolysis in subcutaneous white adipose tissue [53]. However, adverse responses, such as increased heart rate and systolic blood pressure, accompany mirabegron treatment [54]. Importantly, there are no reports of weight loss with mirabegron treatment over periods ranging from 4 to 10 weeks [52,53,54].

Our clinical trial demonstrated that MLR-1023 at 100 mg/day was well-tolerated. Compared to the placebo, MLR1023 reduced FPG and produced weight loss. A statistically significant reduction in body weight was achieved in four weeks, and progressively declined further one week after treatment was stopped. The mean half-life for MLR-1023 (100 mg) in humans is 0.61 h [15]. In rodents, the half-life of MLR-1023 is similar, but the blood-glucose-lowering effect lasts for over 240 min [13]. These data suggest that MLR-1023 induces a durable program which would provide clinically meaningful weight loss over a longer treatment period than four weeks.

In HASCs treated with MLR-1023, we observed the induction of the thermogenic program. Accordingly, UCP1, ATGL, CPT1β, and GLUT4 were upregulated, without any increase in β1AR, which is the most abundant adrenergic receptor in human adipose tissue [35,36]. However, the expression of TRPM8, which is the cold sensor that transduces cold stimuli in the somatosensory neurons, was upregulated. Lyn kinase, which is a Src-related nonreceptor-linked tyrosine kinase, is expressed in sensory neurons; TRPM8 activity is potentiated by tyrosine phosphorylation [55,56]. In human adipocytes TRPM8 is present [29]. Thus, the mechanism of action for TRPM8-mediated induction of the thermogenic program in HASCs following treatment with the Lyn kinase activator, MLR-1023, is likely through tyrosine phosphorylation and the activation of TRPM8.

The increase in thermogenesis was evident in the reduction of body weight in high-fat-fed CD1/ICR mice treated with MLR-1023 without affecting food intake. Brown/beige phenotypes of fat are major thermogenic tissues that protect animals against the adverse metabolic outcomes of a high-fat diet. Therefore, increasing the development and activation of beige fat is a safe and effective strategy to overcome obesity and its comorbidities [57]. TRPM8 is also expressed in mouse brown adipose tissue, and its activation by the cooling agent menthol induces an increase in UCP1 expression through the Ca^2+^-dependent activation of protein kinase A (PKA), rather than adrenergic stimulation [26]. The activation of PKA stimulates lipolysis, and the fatty acids generated are the thermogenic substrates [58]. It is generally accepted that fatty acids or their derivatives are involved in the activation of UCP1 [59].

We observed a reduction in brown, axial, epidydimal, inguinal, and renal fat masses in mice treated with MLR-1023. Consistent with the reduction in fat masses, we observed a reduction in leptin concentrations. Leptin secretion has an evolutionary basis and is designed to maintain the relative constancy of adipose tissue mass by stimulating appetite as fat stores decline [60]. Weight loss is accompanied by a percent decrease in resting energy expenditure that exceeds the percent reduction in body weight. The decline in energy expenditure promotes an increase in food intake [61]. However, we observed no change in food intake despite the reduction in body weight. These data lend further support to the notion that MLR-1023 induces a thermogenic program that increases energy expenditure. Furthermore, the process appears to override the drive to eat that would be expected with the decline in circulating leptin concentrations [60].

In human white adipocytes, the activation of TRPM8 by menthol induces a dose-dependent increase in Ca^2+^ and UCP1 expression together with an increase in glucose uptake and mitochondrial energy production. These changes are accompanied by the appearance of morphological characteristics that are typical of thermogenically active adipocytes [29]. We observed that the treatment of HASCSs with the combination of MLR-1023 and cold stimulation (exposure to 26 °C) produced a robust increase in the expression of UCP1. Moreover, the expression of PRKAR2β increased in HASCs treated with MLR-1023 and cold stimulation, suggesting the activation of thermogenesis through the PKA rather than the adrenergic pathway [26].

Low rates of lipolysis and expression of PRKAR2β, which is the PKA subunit involved in energy balance, are linked to inefficient lipolysis and a predisposition to weight gain as well as impaired glucose metabolism in humans [37]. Beige fat appears to play an important role in glucose homeostasis that goes beyond the dissipation of stored fat as heat [48], and we observed an increase in the expression of UCP1, GLUT4, and adiponectin in HASCs treated with MLR-1023 and menthol. Adiponectin sensitizes peripheral tissues to insulin and is primarily produced by adipocytes; paradoxically, circulating levels are reduced in obese individuals [62].

The causal relationship between increased adiponectin and insulin sensitivity is, in part, mediated by an increase in fat oxidation and a reduction in adipose tissue mass [63,64]. Therefore, we examined the effects on glucose metabolism in diabetic mice treated chronically with the combination of MLR-1023 and menthol. As previously demonstrated, MLR-1023 reduced the glycemic response [12,13]. We found that in combination with menthol, MLR-1023 produced a persistently significant reduction in blood glucose that lasted over two weeks after the treatment ended, whereas the effect of MLR-1023 alone lasted for one week after the treatment ended. Menthol is a food component that is commonly consumed, and MLR-1023 has a proven safety profile in humans. Thus, the amplification of the thermogenic program by MLR-1023 and menthol, which occurs in the absence of adrenergic activation, will likely be well-tolerated, and bears investigation in a clinical trial.

## 4. Materials and Methods

### 4.1. Materials

MLR-1023 was synthesized according to previously developed synthetic methods [65] at the Advanced Synthesis Group (Newark, DE, USA). Aviva glucose test strips and monitors were from Roche Diagnostics (Nutley, NJ, USA). Hyclone™ Dulbecco’s Modified Eagle Medium/Ham’s F12 (DMEM/F12 1:1) and Gibco™ fetal bovine serum (FBS) were purchased from ThermoFisher Scientific (Waltham, MA, USA), and rosiglitazone was purchased from AK Scientific (Union City, CA, USA). All other compounds and reagents were obtained from Sigma-Aldrich (St. Louis, MO, USA), unless otherwise stated.

### 4.2. Animal Studies

All experiments were conducted in accordance with the National Institutes of Health regulations of animal care covered in the Principles of Laboratory Animal Care (National Institutes of Health, 2011), and were approved by the Institutional Animal Care and Use Committee. CD1-ICR male mice that were eight weeks of age (Ace Animals, Boyertown, PA, USA) were used in the studies of food intake and body weight. Six-week-old (at the start of the study) db/db male mice (BKS Cg- + Leprdb/ + Leprdb/OlaHsd) were used in the study to measure fasting plasma glucose (Harlan Laboratories, Indiana, IN, USA). Mice were given free access to food and water. Animals were kept on a 12:12 h light/dark cycle and were maintained on a standard diet. The standard diet was Harlan chow (2016 Teklad Global 16% Protein Rodent Diet; Harlan Laboratories, Indianapolis, Indiana, IN, USA).

### 4.3. Drug Administration 

MLR-1023 was formulated in 2-hydroxypropyl-beta-cyclodextrin (20%). MLR-1023 was administered intraperitoneally (IP), and menthol was administered by oral gavage at dose volumes of 5 mL/kg.

### 4.4. Body Weight and Food Intake Measurements

The CD1-ICR mice were eight weeks of age when the study started. Prior to the initiation of the study, mice were fasted for 24 h. In this study, mice were fed a “Western Diet” that was designed to approximate the “typical” human diet of North America and Europe (Research Diets; New Brunswick, NJ, USA; Western Diet composition). The Western Diet contained 21% of its energy content from fat, compared to 4% from fat in normal chow. MLR-1023 at 30 mg/kg and vehicle (saline) were administered IP twice daily for 30 days. Mice were weighed daily from the start of the 24 h fasting period. Food intake was monitored every one to four days (Figure 5B).

### 4.5. Leptin Measurements and Fat Pad Dissection

Mice were bled by retro-orbital eye bleeds on days 7 and 14 after the initiation of the study. On the day of the retro-orbital eye bleed, mice were dosed once with a full dose one hour prior to the bleed. Leptin levels were determined on days 7 and 14 by ELISA (R&D Systems, Minneapolis, Minnesota, MN, USA), as per directions. Fat pads were dissected at the end of the study (day 31) and subsequently weighed and frozen. Brown, inguinal, axial, mesenteric, renal, and epididymal fat pads were dissected and weighed.

### 4.6. Fasting Plasma Glucose Measurement

Six-week-old db/db mice were treated with MLR-1023, the vehicle, or MLR-1023 and menthol once daily. MLR-1023 (100 mg/kg) was administered IP and menthol was administered by oral gavage at 100 mg/kg for 56 days. Fasting plasma glucose was measured for an additional 28 days after treatment ended. Lean mice (BKS Cg) were similarly treated with the vehicle. Mice were weighed weekly during the study. Fasting plasma glucose levels were measured before the first administration of the test compounds. After the initiation of treatment, blood glucose levels were measured weekly before that day’s compounds were administrated (24 h after the previous administration). For measurements of FPG, mice were fasted for six hours and blood glucose levels were measured using Glucocard Vital glucometers (Arkray, Minneapolis, MN, USA). Blood (<5 µL) was acquired from a tail snip and directly applied to a glucose test strip.

### 4.7. Human Adipocyte Cell Culture Studies

Human-adipose-derived stem cells from overweight and obese female donors were purchased from LaCell, LLC (New Orleans, LA, USA), or isolated from lipoaspirate waste donated post-surgery from women with a body mass index (BMI) ranging from 27 to 36 kg/m^2^, using methods that have been previously described [66]. The cell culture methods have also been previously described [67]. Adipocytes were treated with MLR-1023, menthol, or the combination of MLR-1023 and menthol for seven days in an adipocyte maintenance medium with heat-inactivated serum before RNA or protein were isolated from adipocyte cultures. MLR-1023 was diluted to the final concentration from a 20 mM DMSO stock solution, and menthol was diluted from a 10 mM stock solution in water.

### 4.8. Total RNA Extraction and Quantitative Real-Time Polymerase Chain Reaction (qRT-PCR)

Total RNA was extracted from cells using Tri Reagent (Sigma, Lt. Louis, Missouri, MO, USA) and purified with RNeasy (Qiagen, Germantown, Maryland, MD, USA) into nuclease-free water containing RNAsecure Reagent (Thermo Fisher Scientific, Waltham, MA, USA).

Reverse transcriptase and PCR were conducted in one reaction with the reverse PCR primer priming cDNA synthesis, as we previously described [67]. Primer–probe sequences for UCP1, GLUT4, ATGL, adiponectin, and ribosomal RPL13A are provided in our previously published study [68]. RPL13A was used to normalize for total RNA in each sample. Additional primer and probe oligonucleotide sets used in this study are: TRPM8, shown in a 5′ to 3′ orientation, forward GAGACACCAAGAACTGGAAGAT reverse AGGTGAAGAACGCCACATAG probe TTGGTGGGCTGTGGCTTTGTATCA; predesigned human primer and probe sets from Thermofisher Scientific are ADRB1 Hs02330048_s1 and PRKAR2B Hs01036963_m1.

### 4.9. Immunoblot Analysis

Whole-cell protein was solubilized from cells with a RIPA buffer that contained protease and phosphatase inhibitors (Cell Signaling Technologies, Danvers, MA, USA). Proteins were separated using Precast 7.5% TGX SDS-PAGE gels (BioRad, Hercules, CA, USA) and transferred to nitrocellulose membranes, as we previously described [68]. Nitrocellulose membranes were probed overnight at 4 °C with primary antibodies against adiponectin (Santa-Cruz 136131) or β-Actin (Sigma A5316). HRP-linked anti-mouse (AP130P, Sigma) was used to detect proteins. Proteins were visualized by chemiluminescence (Western Lightning Plus-ECL, PerkinElmer, Waltham, MA, USA).

### 4.10. Clinical Trial

The study was a randomized double-blinded placebo-controlled parallel-arm phase II dose-finding trial to investigate the effect of MLR-1023 on body weight in adults with type 2 diabetes. Subjects were between 18 and 75 years old, on a diet and exercise regimen, and were naïve to glucose-lowering medications or were using (but had discontinued) metformin six months prior to enrollment. The study was conducted in the US and South Korea [15]. In this paper, we report the results from the 100 mg once-daily dose and placebo groups of the US cohort. The study was approved by the Institutional Review Board of each study site, written informed consent was obtained, and the trial was registered with ClinicalTrials.gov, identifier: NCT02317796.

### 4.11. Statistical Analysis

All data were analyzed by one-way analysis of variance or *t*-tests. A mixed model analysis was used to test for differences between groups in the clinical trial and in the analyses of FPG.

## 5. Conclusions

MLR-1023 induces a thermogenic program, reduces body weight in humans, and prevents weight gain in mice fed a high-fat diet, without adrenergic activation. In combination with menthol, the effect of MLR-1023 on thermogenesis is potentiated in human adipocytes, while in mice the glucose-lowering effect is increased. Despite novel treatments, such as sodium glucose transporter 2 (SGLT2) inhibitors and glucagon-like-peptide-1 (GLP-1) receptor agonists, the fraction of patients with well-controlled diabetes has not risen appreciably [16]. The reasons for this could in part be related to the high costs of medications or unwanted side effects. The multiple sites of action of these medications reflect the complex regulation of blood glucose, and highlight the need for developing glucose-lowering drugs that provide protective effects beyond glycemic control. Medications that target glucose-lowering mechanisms and do not induce adverse effects will likely overcome patients’ antipathy and enhance compliance with medication regimens [16]. By promoting the conversion of white adipocytes into a durable energy-expending phenotype without the adverse effects of sympathetic stimulation, the combination of the insulin sensitizer MLR-1023 and menthol offers benefits beyond lowering blood glucose.

## Figures and Tables

**Table 1:** Baseline characteristics of subjects. Values are mean ± SD.

	MLR-1023 100 mg QD	Placebo
Gender		
Female	8	6
Male	12	15
Age (years)	57.45 ± 1.89	60.24 ± 1.71
Weight (kg)	90.73 ± 3.29	83.88 ± 3.93
BMI (kg/m^2^)	32.54 ± 0.83	29.35 ± 0.84

## Data Availability

The data is contained within the article.

## References

[1] IDF International Diabetes Federation: Diabetes Facts and Figures. https://idf.org/aboutdiabetes/what-is-diabetes/facts-figures.html.

[2] CDC (2020). National Diabetes Statistics Report. https://www.cdc.gov/diabetes/data/statistics-report/index.html.

[3] Zhu L., She Z.G., Cheng X., Qin J.J., Zhang X.J., Cai J., Lei F., Wang H., Xie J., Wang W. (2020). Association of Blood Glucose Control and Outcomes in Patients with COVID-19 and Pre-existing Type 2 Diabetes. Cell Metab..

[4] Nalbandian A., Sehgal K., Gupta A., Madhavan M.V., McGroder C., Stevens J.S., Cook J.R., Nordvig A.S., Shalev D., Sehrawat T.S. (2021). Post-acute COVID-19 syndrome. Nat. Med..

[5] Nathan D.M., Buse J.B., Davidson M.B., Ferrannini E., Holman R.R., Sherwin R., Zinman B., American Diabetes Association, European Association for the Study of Diabetes (2009). Medical management of hyperglycemia in type 2 diabetes: A consensus algorithm for the initiation and adjustment of therapy: A consensus statement of the American Diabetes Association and the European Association for the Study of Diabetes. Diabetes Care.

[6] Nathan D.M., Buse J.B., Kahn S.E., Krause-Steinrauf H., Larkin M.E., Staten M., Wexler D., Lachin J.M., Group G.S.R. (2013). Rationale and design of the glycemia reduction approaches in diabetes: A comparative effectiveness study (GRADE). Diabetes Care.

[7] Kahn S.E., Haffner S.M., Heise M.A., Herman W.H., Holman R.R., Jones N.P., Kravitz B.G., Lachin J.M., O’Neill M.C., Zinman B. (2006). Glycemic durability of rosiglitazone, metformin, or glyburide monotherapy. N. Engl. J. Med..

[8] Ekstrom N., Svensson A.M., Miftaraj M., Andersson Sundell K., Cederholm J., Zethelius B., Eliasson B., Gudbjornsdottir S. (2015). Durability of oral hypoglycemic agents in drug naive patients with type 2 diabetes: Report from the Swedish National Diabetes Register (NDR). BMJ Open Diabetes Res. Care.

[9] Turner R.C., Cull C.A., Frighi V., Holman R.R. (1999). Glycemic control with diet, sulfonylurea, metformin, or insulin in patients with type 2 diabetes mellitus: Progressive requirement for multiple therapies (UKPDS 49): UK Prospective Diabetes Study (UKPDS) Group. JAMA.

[10] Brown J.B., Conner C., Nichols G.A. (2010). Secondary failure of metformin monotherapy in clinical practice. Diabetes Care.

[11] Lipinski C.A., Reaume A.G. (2020). High throughput in vivo phenotypic screening for drug repurposing: Discovery of MLR-1023 a novel insulin sensitizer and novel Lyn kinase activator with clinical proof of concept. Bioorg. Med. Chem..

[12] Ochman A.R., Lipinski C.A., Handler J.A., Reaume A.G., Saporito M.S. (2012). The Lyn kinase activator MLR-1023 is a novel insulin receptor potentiator that elicits a rapid-onset and durable improvement in glucose homeostasis in animal models of type 2 diabetes. J. Pharmacol. Exp. Ther..

[13] Saporito M.S., Ochman A.R., Lipinski C.A., Handler J.A., Reaume A.G. (2012). MLR-1023 is a potent and selective allosteric activator of Lyn kinase in vitro that improves glucose tolerance in vivo. J. Pharmacol. Exp. Ther..

[14] Muller G., Wied S., Frick W. (2000). Cross talk of pp125(FAK) and pp59(Lyn) non-receptor tyrosine kinases to insulin-mimetic signaling in adipocytes. Mol. Cell. Biol..

[15] Lee M.K., Kim S.G., Watkins E., Moon M.K., Rhee S.Y., Frias J.P., Chung C.H., Lee S.H., Block B., Cha B.S. (2020). A novel non-PPARgamma insulin sensitizer: MLR-1023 clinicalproof-of-concept in type 2 diabetes mellitus. J. Diabetes Complicat..

[16] Nauck M.A., Wefers J., Meier J.J. (2021). Treatment of type 2 diabetes: Challenges, hopes, and anticipated successes. Lancet Diabetes Endocrinol..

[17] Cariou B., Charbonnel B., Staels B. (2012). Thiazolidinediones and PPARgamma agonists: Time for a reassessment. Trends Endocrinol. Metab. TEM.

[18] Lago R.M., Singh P.P., Nesto R.W. (2007). Congestive heart failure and cardiovascular death in patients with prediabetes and type 2 diabetes given thiazolidinediones: A meta-analysis of randomised clinical trials. Lancet.

[19] Erdmann E., Charbonnel B., Wilcox R.G., Skene A.M., Massi-Benedetti M., Yates J., Tan M., Spanheimer R., Standl E., Dormandy J.A. (2007). Pioglitazone use and heart failure in patients with type 2 diabetes and preexisting cardiovascular disease: Data from the PROactive study (PROactive 08). Diabetes Care.

[20] Kernan W.N., Viscoli C.M., Furie K.L., Young L.H., Inzucchi S.E., Gorman M., Guarino P.D., Lovejoy A.M., Peduzzi P.N., Conwit R. (2016). Pioglitazone after Ischemic Stroke or Transient Ischemic Attack. N. Engl. J. Med..

[21] Lewis J.D., Ferrara A., Peng T., Hedderson M., Bilker W.B., Quesenberry C.P., Vaughn D.J., Nessel L., Selby J., Strom B.L. (2011). Risk of bladder cancer among diabetic patients treated with pioglitazone: Interim report of a longitudinal cohort study. Diabetes Care.

[22] Dormandy J.A., Charbonnel B., Eckland D.J., Erdmann E., Massi-Benedetti M., Moules I.K., Skene A.M., Tan M.H., Lefebvre P.J., Murray G.D. (2005). Secondary prevention of macrovascular events in patients with type 2 diabetes in the PROactive Study (PROspective pioglitAzone Clinical Trial in macroVascular Events): A randomised controlled trial. Lancet.

[23] Muller G., Jung C., Wied S., Welte S., Jordan H., Frick W. (2001). Redistribution of glycolipid raft domain components induces insulin-mimetic signaling in rat adipocytes. Mol. Cell. Biol..

[24] Ye R., Holland W.L., Gordillo R., Wang M., Wang Q.A., Shao M., Morley T.S., Gupta R.K., Stahl A., Scherer P.E. (2014). Adiponectin is essential for lipid homeostasis and survival under insulin deficiency and promotes beta-cell regeneration. eLife.

[25] Qi Y., Takahashi N., Hileman S.M., Patel H.R., Berg A.H., Pajvani U.B., Scherer P.E., Ahima R.S. (2004). Adiponectin acts in the brain to decrease body weight. Nat. Med..

[26] Ma S., Yu H., Zhao Z., Luo Z., Chen J., Ni Y., Jin R., Ma L., Wang P., Zhu Z. (2012). Activation of the cold-sensing TRPM8 channel triggers UCP1-dependent thermogenesis and prevents obesity. J. Mol. Cell Biol..

[27] Tajino K., Matsumura K., Kosada K., Shibakusa T., Inoue K., Fushiki T., Hosokawa H., Kobayashi S. (2007). Application of menthol to the skin of whole trunk in mice induces autonomic and behavioral heat-gain responses. Am. J. Physiol. Regul. Integr. Comp. Physiol..

[28] Xu L., Han Y., Chen X., Aierken A., Wen H., Zheng W., Wang H., Lu X., Zhao Z., Ma C. (2020). Molecular mechanisms underlying menthol binding and activation of TRPM8 ion channel. Nat. Commun..

[29] Rossato M., Granzotto M., Macchi V., Porzionato A., Petrelli L., Calcagno A., Vencato J., De Stefani D., Silvestrin V., Rizzuto R. (2014). Human white adipocytes express the cold receptor TRPM8 which activation induces UCP1 expression, mitochondrial activation and heat production. Mol. Cell. Endocrinol..

[30] Smith U., Kahn B.B. (2016). Adipose tissue regulates insulin sensitivity: Role of adipogenesis, de novo lipogenesis and novel lipids. J. Intern. Med..

[31] Sidossis L., Kajimura S. (2015). Brown and beige fat in humans: Thermogenic adipocytes that control energy and glucose homeostasis. J. Clin. Investig..

[32] Mottillo E.P., Balasubramanian P., Lee Y.H., Weng C., Kershaw E.E., Granneman J.G. (2014). Coupling of lipolysis and de novo lipogenesis in brown, beige, and white adipose tissues during chronic beta3-adrenergic receptor activation. J. Lipid Res..

[33] Bartesaghi S., Hallen S., Huang L., Svensson P.A., Momo R.A., Wallin S., Carlsson E.K., Forslow A., Seale P., Peng X.R. (2015). Thermogenic activity of UCP1 in human white fat-derived beige adipocytes. Mol. Endocrinol..

[34] Bautista D.M., Siemens J., Glazer J.M., Tsuruda P.R., Basbaum A.I., Stucky C.L., Jordt S.E., Julius D. (2007). The menthol receptor TRPM8 is the principal detector of environmental cold. Nature.

[35] Mattsson C.L., Csikasz R.I., Chernogubova E., Yamamoto D.L., Hogberg H.T., Amri E.Z., Hutchinson D.S., Bengtsson T. (2011). beta(1)-Adrenergic receptors increase UCP1 in human MADS brown adipocytes and rescue cold-acclimated beta(3)-adrenergic receptor-knockout mice via nonshivering thermogenesis. Am. J. Physiol. Endocrinol. Metab..

[36] Riis-Vestergaard M.J., Richelsen B., Bruun J.M., Li W., Hansen J.B., Pedersen S.B. (2020). Beta-1 and Not Beta-3 Adrenergic Receptors May Be the Primary Regulator of Human Brown Adipocyte Metabolism. J. Clin. Endocrinol. Metab..

[37] Arner P., Andersson D.P., Backdahl J., Dahlman I., Ryden M. (2018). Weight Gain and Impaired Glucose Metabolism in Women Are Predicted by Inefficient Subcutaneous Fat Cell Lipolysis. Cell Metab..

[38] Faulds G., Ryden M., Ek I., Wahrenberg H., Arner P. (2003). Mechanisms behind lipolytic catecholamine resistance of subcutaneous fat cells in the polycystic ovarian syndrome. J. Clin. Endocrinol. Metab..

[39] American Diabetes A. (2021). 9. Pharmacologic Approaches to Glycemic Treatment: Standards of Medical Care in Diabetes-2021. Diabetes Care.

[40] Flory J., Lipska K. (2019). Metformin in 2019. JAMA.

[41] Rosenstock J., Kahn S.E., Johansen O.E., Zinman B., Espeland M.A., Woerle H.J., Pfarr E., Keller A., Mattheus M., Baanstra D. (2019). Effect of Linagliptin vs Glimepiride on Major Adverse Cardiovascular Outcomes in Patients With Type 2 Diabetes: The CAROLINA Randomized Clinical Trial. JAMA.

[42] Kadowaki T., Wang G., Rosenstock J., Yabe D., Peng Y., Kanasaki K., Mu Y., Mattheus M., Keller A., Okamura T. (2021). Effect of linagliptin, a dipeptidyl peptidase-4 inhibitor, compared with the sulfonylurea glimepiride on cardiovascular outcomes in Asians with type 2 diabetes: Subgroup analysis of the randomized CAROLINA(R) trial. Diabetol. Int..

[43] Chan J.C.N., Lim L.L., Wareham N.J., Shaw J.E., Orchard T.J., Zhang P., Lau E.S.H., Eliasson B., Kong A.P.S., Ezzati M. (2021). The Lancet Commission on diabetes: Using data to transform diabetes care and patient lives. Lancet.

[44] Ghorbani M., Himms-Hagen J. (1997). Appearance of brown adipocytes in white adipose tissue during CL 316,243-induced reversal of obesity and diabetes in Zucker fa/fa rats. Int. J. Obes. Relat. Metab. Disord..

[45] Granneman J.G., Li P., Zhu Z., Lu Y. (2005). Metabolic and cellular plasticity in white adipose tissue I: Effects of beta3-adrenergic receptor activation. Am. J. Physiol. Endocrinol. Metab..

[46] Himms-Hagen J., Melnyk A., Zingaretti M.C., Ceresi E., Barbatelli G., Cinti S. (2000). Multilocular fat cells in WAT of CL-316243-treated rats derive directly from white adipocytes. Am. J. Physiol. Cell Physiol..

[47] Petrovic N., Walden T.B., Shabalina I.G., Timmons J.A., Cannon B., Nedergaard J. (2010). Chronic peroxisome proliferator-activated receptor gamma (PPARgamma) activation of epididymally derived white adipocyte cultuRes. reveals a population of thermogenically competent, UCP1-containing adipocytes molecularly distinct from classic brown adipocytes. J. Biol. Chem..

[48] Kajimura S., Spiegelman B.M., Seale P. (2015). Brown and Beige Fat: Physiological Roles beyond Heat Generation. Cell Metab..

[49] Long J.Z., Svensson K.J., Tsai L., Zeng X., Roh H.C., Kong X., Rao R.R., Lou J., Lokurkar I., Baur W. (2014). A smooth muscle-like origin for beige adipocytes. Cell Metab..

[50] Lowell B.B., Spiegelman B.M. (2000). Towards a molecular understanding of adaptive thermogenesis. Nature.

[51] Moisan A., Lee Y.K., Zhang J.D., Hudak C.S., Meyer C.A., Prummer M., Zoffmann S., Truong H.H., Ebeling M., Kiialainen A. (2015). White-to-brown metabolic conversion of human adipocytes by JAK inhibition. Nat. Cell Biol..

[52] Finlin B.S., Memetimin H., Confides A.L., Kasza I., Zhu B., Vekaria H.J., Harfmann B., Jones K.A., Johnson Z.R., Westgate P.M. (2018). Human adipose beiging in response to cold and mirabegron. JCI Insight.

[53] Finlin B.S., Memetimin H., Zhu B., Confides A.L., Vekaria H.J., El Khouli R.H., Johnson Z.R., Westgate P.M., Chen J., Morris A.J. (2020). The beta3-adrenergic receptor agonist mirabegron improves glucose homeostasis in obese humans. J. Clin. Investig..

[54] O’Mara A.E., Johnson J.W., Linderman J.D., Brychta R.J., McGehee S., Fletcher L.A., Fink Y.A., Kapuria D., Cassimatis T.M., Kelsey N. (2020). Chronic mirabegron treatment increases human brown fat, HDL cholesterol, and insulin sensitivity. J. Clin. Investig..

[55] McKemy D.D., Neuhausser W.M., Julius D. (2002). Identification of a cold receptor reveals a general role for TRP channels in thermosensation. Nature.

[56] Manolache A., Selescu T., Maier G.L., Mentel M., Ionescu A.E., Neacsu C., Babes A., Szedlacsek S.E. (2020). Regulation of TRPM8 channel activity by Src-mediated tyrosine phosphorylation. J. Cell. Physiol..

[57] Wang W., Seale P. (2016). Control of brown and beige fat development. Nat. Rev. Mol. Cell Biol..

[58] Fredriksson J.M., Thonberg H., Ohlson K.B., Ohba K., Cannon B., Nedergaard J. (2001). Analysis of inhibition by H89 of UCP1 gene expression and thermogenesis indicates protein kinase A mediation of beta(3)-adrenergic signalling rather than beta(3)-adrenoceptor antagonism by H89. Biochim. Biophys. Acta.

[59] Cannon B., Nedergaard J. (2004). Brown adipose tissue: Function and physiological significance. Physiol. Rev..

[60] Friedman J.M. (2019). Leptin and the endocrine control of energy balance. Nat. Metab..

[61] Leibel R.L., Rosenbaum M., Hirsch J. (1995). Changes in energy expenditure resulting from altered body weight. N. Engl. J. Med..

[62] Turer A.T., Khera A., Ayers C.R., Turer C.B., Grundy S.M., Vega G.L., Scherer P.E. (2011). Adipose tissue mass and location affect circulating adiponectin levels. Diabetologia.

[63] Kizer J.R. (2013). A tangled threesome: Adiponectin, insulin sensitivity, and adiposity: Can Mendelian randomization sort out causality?. Diabetes.

[64] Lihn A.S., Pedersen S.B., Richelsen B. (2005). Adiponectin: Action, regulation and association to insulin sensitivity. Obes. Rev..

[65] Lipinski C.A., Stam J.G., Pereira J.N., Ackerman N.R., Hess H.J. (1980). Bronchodilator and antiulcer phenoxypyrimidinones. J. Med. Chem..

[66] Li J., Curley J.L., Floyd Z.E., Wu X., Halvorsen Y.D.C., Gimble J.M. (2018). Isolation of Human Adipose-Derived Stem Cells from Lipoaspirates. Methods Mol. Biol..

[67] Rebello C.J., Greenway F.L., Johnson W.D., Ribnicky D., Poulev A., Stadler K., Coulter A.A. (2017). Fucoxanthin and Its Metabolite Fucoxanthinol Do Not Induce Browning in Human Adipocytes. J. Agric. Food Chem..

[68] Rebello C.J., Greenway F.L., Lau F.H., Lin Y., Stephens J.M., Johnson W.D., Coulter A.A. (2019). Naringenin Promotes Thermogenic Gene Expression in Human White Adipose Tissue. Obesity (Silver Spring).

